# Impact of Substance Use Disorder Pharmacotherapy on Executive Function: A Narrative Review

**DOI:** 10.3389/fpsyt.2019.00098

**Published:** 2019-03-01

**Authors:** Kevin Butler, Bernard Le Foll

**Affiliations:** ^1^Translational Addiction Research Laboratory, Centre for Addiction and Mental Health, University of Toronto, Toronto, ON, Canada; ^2^Alcohol Research and Treatment Clinic, Acute Care Program, Centre for Addiction and Mental Health, Toronto, ON, Canada; ^3^Campbell Family Mental Health Research Institute, Centre for Addiction and Mental Health, Toronto, ON, Canada; ^4^Department of Family and Community Medicine, University of Toronto, Toronto, ON, Canada; ^5^Department of Pharmacology and Toxicology, University of Toronto, Toronto, ON, Canada; ^6^Division of Brain and Therapeutics, Department of Psychiatry, University of Toronto, Toronto, ON, Canada; ^7^Institute of Medical Sciences, University of Toronto, Toronto, ON, Canada

**Keywords:** addiction, cognitive enhancement, cognitive flexibility, executive function, inhibitory control, pharmacotherapy, substance use disorder, working memory

## Abstract

Substance use disorders are chronic, relapsing, and harmful conditions characterized by executive dysfunction. While there are currently no approved pharmacotherapy options for stimulant and cannabis use disorders, there are several evidence-based options available to help reduce symptoms during detoxification and aid long-term cessation for those with tobacco, alcohol and opioid use disorders. While these medication options have shown clinical efficacy, less is known regarding their potential to enhance executive function. This narrative review aims to provide a brief overview of research that has investigated whether commonly used pharmacotherapies for these substance use disorders (nicotine, bupropion, varenicline, disulfiram, acamprosate, nalmefene, naltrexone, methadone, buprenorphine, and lofexidine) effect three core executive function components (working memory, inhibitory control and cognitive flexibility). While pharmacotherapy-induced enhancement of executive function may improve cessation outcomes in dependent populations, there are limited and inconsistent findings regarding the effects of these medications on executive function. We discuss possible reasons for the mixed findings and suggest some future avenues of work that may enhance the understanding of addiction pharmacotherapy and cognitive training interventions and lead to improved patient outcomes.

## Introduction

Substance use disorders are chronic, relapsing conditions (1) with huge costs to the individual and to society. For example, using data from 2015, Peacock et al. (2) estimate global prevalence of past 30 day heavy alcohol use, daily smoking and past year opioid use at 18.4, 15.2, and 0.37%, respectively and they estimate disease burden with the number of disability-adjusted life years (the number of years lost due to ill-health, disability, or early death) as 170.9 million, 85.0 million and 27.8 million for tobacco smoking, alcohol, and illicit drug use, respectively. Indeed, alcohol, heroin, and tobacco have previously been rated amongst the most harmful misused drugs when considering harms to both the individual and to others (3).

There are several psychological/behavioral treatments available for substance use disorders [for a brief overview see McGovern and carroll (4)]. While there are no approved pharmacotherapies for stimulant and cannabis use disorders, evidence-based pharmacological agents are available for tobacco, alcohol and opioid use disorders (TUD, AUD, and OUD, respectively). Medications currently approved for these disorders include nicotine replacement therapy, bupropion, varenicline (for TUD), disulfiram, acamprosate, naltrexone, nalmefene (for AUD), methadone, buprenorphine, naltrexone, and lofexidine (for OUD). While previous research has found these drugs to be efficacious, relapse in drug dependence is 40–60% (5) suggesting efficacy is limited and that there is room for improvement in the management of addictions.

Cognitive processes may be important targets for the treatment of substance use disorders (6, 7). In particular, executive dysfunction is considered a hallmark of addiction (8, 9) and may represent a good transdiagnostic target across addictive disorders. Impairments in executive function may contribute to the initiation and maintenance of problematic drug use. For instance, executive function at an early age predicts subsequent substance use (10) and performance comparisons across drug users, non-addicted family members and healthy controls suggest that deficits in executive function may be a cognitive endophenotype associated with drug dependence vulnerability (11, 12). Executive function deficits are also related to relapse, worse clinical outcomes and poor treatment adherence (13–19) with exacerbation of executive function impairments observed during early abstinence which may contribute to relapse (20–22).

While the clinical efficacy of approved pharmacotherapy for TUD, AUD, and OUD is recognized, there has been far less research conducted on the cognitive effects of these medications (23) despite potential cognitive enhancement effects contributing to clinical efficacy. Therefore, the goal of this review is to provide a brief and selective, narrative summary of the evidence examining the impact of nicotine, bupropion, varenicline, disulfiram, acamprosate, nalmefene, naltrexone, methadone, buprenorphine, and lofexidine on executive function. We do not include medications used off-label to treat substance use disorders due to the wide-range of off-label prescribing practices, limited, or inconsistent evidence for clinical efficacy and because we cannot be certain which of these medications will continue to look effective as the evidence base for them increases. This review complements the recent systematic review that investigated general cognitive effects of pharmacotherapy for substance use disorders (23). While this earlier review provides a good overview of the cognitive impact of substance use disorder medication, its discussion of the impact on executive function could be considered limited by the general approach to cognition that has been taken. The current review fractionates executive function and focuses on working memory, inhibitory control, and cognitive flexibility as there is general agreement that these are the three core executive function components and that other higher-order executive functions such as decision-making, planning, problem-solving, and reasoning may require these basic components (24, 25). Additionally, the current review also takes a translational approach by including relevant findings from research with non-human animals where human research is scarce or it adds to an understanding of drug effects.

## Pharmacotherapies for Tobacco Use Disorder

### Nicotine

The nicotinic acetylcholine receptor agonist nicotine is used in those with TUD as a replacement therapy where it can be delivered in many forms including chewing gum and adhesive skin patches. When used as an aid to quit smoking or chewing tobacco, nicotine replacement therapy helps to manage withdrawal symptoms associated with cessation and can increase the rate of quitting by up to 50–70% (26). Both α_4_β_2_ and α_7_ nicotinic receptor subtypes have been implicated in cognitive enhancement (27). Indeed, a considerable amount of evidence exists regarding the cognitive enhancing effects of nicotine. For instance, nicotine can improve some abstinence associated cognitive impairments (28). Additionally, a 2010 meta-analysis suggests that fine motor, alerting attention-accuracy and response time, orienting attention reaction time, short-term episodic memory accuracy, and working memory reaction time are particularly sensitive to enhancement following administration of nicotine (29). Furthermore, because the studies included in this meta-analysis used non-smokers or non-/minimally deprived smokers the cognitive enhancement is unlikely to be driven by relief from withdrawal but, instead, represents true cognitive enhancement.

However, reported effects of nicotine on working memory are far from consistent. Animal work suggests that working memory (radial-arm maze) performance is improved by nicotine administration (30) and that methamphetamine or ketamine-induced impairments in working memory (radial-arm maze, odor span task) can be improved by nicotine (31, 32). On the other hand, no effect on working memory (digit recall, serial addition/subtraction, n-back task, digit span, spatial span, letter-number sequencing, odor span task) has been seen in human studies that have administered 2 and 4 mg nicotine gum relative to placebo in healthy non-smoking participants (33–35). Another study found that 15 mg nicotine patches improved working memory (n-back task reaction time) in deprived smokers relative to placebo while they had no benefit in healthy non-smokers but instead impaired performance with significantly fewer hits, more misses and false alarms and a trend toward longer reaction times (36). Taken together this suggests that nicotine may improve working memory when there is impaired baseline performance present but has no effect or impairs performance when baseline performance is higher (37).

Nicotine administration has also been found to improve inhibitory control (antisaccade task, errors of commission on a continuous performance task) deficits that are induced by overnight smoking abstinence (38). However, 7 mg nicotine patches do not improve inhibitory control (stop-signal task, go/nogo task, antisaccade task) in healthy non-smokers (39, 40). In contrast to the findings of these studies, several nicotine administration studies in animals have shown that nicotine can induce disinhibition with increased impulsive responding evident across a range of behavioral tasks (41–47). Similarly, acute cigarette smoking may bias responding to more impulsive action and impulsive choices (48, 49). As with the effect of nicotine on working memory, the mixed findings with nicotine apparently able to improve, impair or have no effect on inhibitory control may be due to baseline differences in performance and several previous studies support this idea. For example, nicotine enhances inhibitory control (fewer errors of commission on a continuous performance task) in non-smokers that have low levels of attention but not in those with high levels of attention (50) while in another study, nicotine enhanced inhibitory control (fewer errors of commission on a continuous performance task) in those with a diagnosis of schizophrenia but not in healthy controls (51). Finally, the effect of chronic nicotine exposure on impulsivity in rats may be influenced by baseline levels of impulsivity with nicotine inducing greater impulsive choice in those with lower trait impulsivity (52, 53).

Few studies have examined the effects of nicotine on cognitive flexibility and those which have reveal mixed findings, much as studies assessing working memory and inhibitory control have. Acute cigarette smoking has been shown to impair cognitive flexibility (more intra-dimensional set-shifting errors on an intra-extra dimensional set-shifting task) in high but not low dependent smokers (54) and (greater difficulty integrating reinforcement history on a reversal learning task) relative to never and former smokers (55). Cognitive flexibility has also been shown to be impaired (poorer learning of strategies to complete the task in the Wisconsin Card Sorting test) by 7 mg nicotine patch administration relative to placebo in non-smokers with high but not low levels of attention (50). Nicotine administration at high (18 mg/Kg/day × 4 weeks) but not low dose (6.3 mg/Kg/day × 4 weeks) also impaired cognitive flexibility (increased perseverative responding to previously non-reinforced stimuli in a reversal learning task) in mice (56). Conversely, improvements in cognitive flexibility (attentional set-shifting task) and reversal of nicotine withdrawal-induced impairment in cognitive flexibility (reversal learning task) have both been reported in rats (57, 58). While in another human study, cognitive flexibility (attentional switching on the flexibility of attention test) was not changed by nicotine (59). As with working memory and inhibitory control, mixed findings like these suggest that baseline performance levels may be influential in determining cognitive effects of nicotine. As nicotine can induce dopamine release (60), as smoking does in humans (61), a more biological explanation for the mixed findings reported throughout this section might be that performance and dopamine levels are related such that at optimal dopamine levels executive function performance is at its peak i.e., the inverted “U” curve theory (62, 63). Release of dopamine by nicotine could therefore improve or impair performance depending on initial dopamine levels.

### Bupropion

Used clinically for depression as well as a smoking cessation aid, bupropion is a norepinephrine-dopamine reuptake inhibitor and a nicotinic acetylcholine receptor antagonist. Bupropion reduces the severity of nicotine craving and withdrawal symptoms, its clinical effectiveness as a smoking cessation aid is comparable to nicotine replacement therapy and is independent of its antidepressant effect (64, 65). Symptoms that improve in depressed patients that respond to bupropion include those reflecting cognitive disturbance (66). Indeed, one study in patients with major depressive disorder has shown that while serotonin selective reuptake inhibitor-treated patients show cognitive impairments including worse cognitive flexibility relative to matched healthy controls, bupropion-treated patients had normalized cognitive performance with better cognitive flexibility but with no significant mean difference compared to controls (67). Further, another study in those with major depressive disorder found that 8 weeks of bupropion treatment lead to improvements on tasks requiring cognitive flexibility (Trail Making B), working memory and reasoning [A not B Task; (68)].

Few studies have investigated the effects of bupropion on executive function in smokers and the findings of existing studies have been equivocal. One study in 24 smokers with high interest in quitting reported that working memory (correct response times on an N-Back task) was improved by bupropion compared to placebo on the first day of a quit attempt (69). In contrast, another study in 58 smokers (36 male, 22 female) found that bupropion enhanced working memory (Digit Span task) in females but not males whereas it enhanced inhibitory control (inhibiting choice of immediate rewards over a larger, delayed reward) in males but not females during early abstinence (70). A final study in smokers investigated effects of both abstinence and bupropion on cognitive function in adults with schizophrenia. However, in this study 1 week of abstinence was not associated with deficits in working memory (Digit Span task) and controlling for abstinence status, bupropion wasn't associated with better working memory performance (71). Similar null findings have been observed in healthy participants where working memory (Digit Span task) was not improved by either a single dose (150mg) or 2 weeks repeated administration (150 mg x 6days followed by 300 mg x 8days) of bupropion (72). However, in an animal study there were positive effects of bupropion on inhibitory control. In this study, rats were perinatally exposed to polychlorinated biphenyls thought to decrease medial prefrontal cortical dopamine levels and cause subsequent inhibitory control deficits assessed with a differential reinforcement of low rates of responding (DRL) task. This study showed that bupropion improved inhibitory control performance on the DRL task (73).

### Varenicline

As a partial agonist at α_4_β_2_ nicotinic acetylcholine receptors, varenicline has been found to reduce craving and the pleasurable effects of tobacco and is more effective for smoking cessation than both nicotine replacement therapy and bupropion (74). Varenicline can reverse withdrawal-associated working memory impairment (75). Patterson et al. (75) showed that in abstinent smokers varenicline, vs. placebo, improved reaction times on correct N-back trials with no significant effects on task accuracy. Interestingly, slower correct responses on the N-Back task predict more rapid resumption of smoking during a short period of abstinence in smokers receiving placebo but not varenicline (76). Beyond simply improving withdrawal-associated impairment, varenicline (0.5 mg/day ×3days followed by 1 mg/day × 4days) administered to non-smokers has been shown to also improve working memory performance (77) with a significant positive association found between plasma varenicline levels and visual-spatial working memory in another non-smoker study (78). There are mixed findings regarding working memory performance in studies with other populations for instance varenicline (1 mg/day × 3 days) attenuated withdrawal-associated working memory impairments in smokers with schizophrenia (79) but did not improve working memory in smokers with schizophrenia who are not treatment-seeking and could continue to smoke (80, 81). Mixed working memory findings have also been found with varenicline in human studies with populations that have other substance abuse problems. For example, varenicline has been shown to improve working memory in heavy drinkers; with larger improvements predicting less alcohol-primed *ad libitum* drinking (82), but not in non-treatment seeking methamphetamine dependent participants (83). While an animal study found that varenicline improves working memory in cocaine-experienced monkeys (84). These mixed findings for other substances of abuse and across different species make it difficult to draw firm conclusions regarding varenicline's cognitive impact. However, as described above evidence suggests that there is some cognitive benefit for certain types of abstinent smoker (75).

Studies regarding varenicline effects on inhibitory control are also mixed. For instance, animal studies indicate that varenicline increases premature responding (failure to inhibit a response during a wait period) on a 5-Choice Serial Reaction Time task (85) however, using a similar 3-Choice task, Ohmura et al. (86) demonstrate that this pro-impulsive effect is evident for nicotine-naïve but not nicotine-exposed or nicotine-abstinent animals. In human studies there was no significant effect of varenicline, compared to placebo, on inhibitory control assessed with a stop-signal task in treatment-seeking smokers (87). In contrast, impulsive responding was increased on a stop-signal task by cigarette smoking and by varenicline [albeit to a smaller degree than smoking; (48)]. However, Austin et al. (48) also found that varenicline attenuated smoking-induced impulsive responding. Varenicline has also been found to reduce antisaccadic error rate (an oculomotor measure of disinhibition) in those with schizophrenia/schizoaffective disorder regardless of smoking status (80).

Fewer studies have reported effects of varenicline on cognitive flexibility. Animal studies have provided mixed findings with Gould et al. (84) finding no effect of varenicline on reversal learning (at doses that give maximum improvement in working memory) in rhesus monkeys. However, varenicline reduced ketamine-induced impairments in reversal learning (accuracy and perseverative responding) and improved working memory (accuracy at long delays on a delay match to sample task) in rhesus and pigtail monkeys (88). In studies related more specifically to smoking, varenicline reversed nicotine withdrawal-induced deficits in the number of reversals on a probabilistic reversal learning task administered to rats (58). While in a human study comparing 24 abstinent smokers with 20 non-smokers, impairments on a reversal learning task (increased response shifting with decisions less sensitive to available evidence) found in abstinent smokers were attenuated by varenicline. In addition, decreased mesocorticolimbic activity associated with shifting in abstinent smokers was increased to the level of non-smokers by varenicline (89). It should be noted that as with nicotine, varenicline produces elevation of dopamine (90).

## Pharmacotherapies for Alcohol Use Disorder

### Disulfiram

By inhibiting the enzyme aldehyde dehydrogenase, disulfiram administration leads to acetaldehyde accumulation when alcohol is consumed. This results in an unpleasant reaction consisting of tachycardia, flushing, nausea, and vomiting. This aversion therapy creates the expectancy of negative consequences that are thought to deter alcohol use. Disulfiram is an efficacious treatment in supervised and high compliance open label studies but not blinded studies suggesting that expectancy may be a requirement of clinical effectiveness [for a review and meta-analysis of efficacy see Skinner et al. (91)]. There is evidence that anti-addictive effects may be mediated by an additional mechanism of action. For example, in rats disulfiram reduces drug-induced reinstatement of cocaine seeking via dopamine β-hydroxylase inhibition (92). Similarly reductions in chocolate self-administration and reinstatement of chocolate seeking have also been observed in rats treated with disulfiram (93) and there are reports that it may have potential for treatment of pathological gambling (94, 95) and cocaine dependence (96).

Few studies have investigated disulfiram's cognitive effects (see Pujol et al. (23) for an overview). In terms of executive function, there were no effects of disulfiram on working memory assessed with the Digit Span Test (97). Similarly, Gilman et al. (98) found no group differences on an extensive test battery, including tasks assessing executive function, when comparing 11 alcoholic patients receiving disulfiram and 37 alcoholic patients not receiving the drug. In contrast, disulfiram administration has been shown to improve inhibitory control (by inhibiting preference for immediate gain at the expense of reduced net gain) in rats that were making suboptimal choices but not those whose choices were already optimal (94).

### Acamprosate

Although the precise mechanism of action is not fully understood, acamprosate is thought to correct imbalance in inhibitory and excitatory neurotransmission induced by chronic alcohol exposure (99). Acamprosate has been found to be a safe and efficacious anti-craving and anti-relapse agent (100). There have been limited studies examining acamprosate effects on executive function. The drugs proposed mechanism of action at NMDA receptors suggests acamprosate would have negative effects on learning and memory, indeed previous cognitive work in healthy participants indicates an acamprosate-induced impairment in delayed free recall. However, working memory was unaffected by acamprosate in the same participants (101). Similarly, there was no significant effect of acamprosate on working memory performance of rats in a three-panel runway task. Although, performance (both errors and latency) was better in acamprosate and scopolamine-treated rats compared to when they were administered the muscarinic acetylcholine receptor antagonist alone (102). There have been mixed findings with studies investigating cognitive flexibility. While Ralevski et al. (103) found no significant effects of acamprosate in 23 alcohol-dependent schizophrenic patients on the Wisconsin Card Sorting Test, animal studies suggest that acamprosate reverses chronic alcohol-induced impairments in attentional set-shifting including reducing task perseveration (104). More recent evidence suggests that these cognitive effects may be related to acamprosate's calcium moiety as a sodium salt version of the drug failed to reverse chronic alcohol-induced deficits in cognition (105).

### Nalmefene

Approved in Europe but not in America, nalmefene is an antagonist at μ-opioid and δ-opioid receptors as well as a partial agonist at κ-opioid receptors thus reducing the positive, rewarding effects of acute alcohol consumption. Nalmefene has greater affinity for κ-opioid receptors than naltrexone does (106). Nalmefene also differs from naltrexone in having a longer half-life, greater bioavailability and no observed dose-dependent liver toxicity [see review by Niciu and Arias (107)]. To the best of our knowledge, there has not been any published research investigating the effects of nalmefene on executive function. However, the κ-opioid receptor agonists nalfurafine and U50,488 produce deficits in inhibitory control (DRL, delay-discounting and stop-signal tasks) in mice and rats (108, 109). U50,488 also produces deficits in cognitive flexibility (modified water maze task) in mice that are reversed by the κ-opioid receptor antagonist nor-binaltorphimine (110). Another κ-opioid receptor agonist U69,593 enhances, while nor-binaltorphimine disrupts working memory (Y-maze) in mice (111). Future studies with nalmefene are warranted because these animal studies suggest that modulation of κ-opioid receptors effects executive function which may be beneficial in disorders characterized by executive dysfunction, such as addiction. Studies examining general cognitive effects of nalmefene are also scarce with one report suggesting increases in subjective alertness but no effect on a choice reaction time task (112).

## Pharmacotherapies for Alcohol and Opioid Use Disorders

### Naltrexone

Pharmacologically, naltrexone has greatest affinity for the μ-opioid receptor but is an antagonist at all opioid receptors and it reduces the rewarding effects as well as craving and desire for alcohol and opiates (113, 114). Indeed, mice lacking the μ-opioid receptor do not self-administer alcohol (115). There have been a small number of human and animal studies examining the effects of naltrexone on executive function. After 8 weeks of administration, Hatsukami et al. (116) found no significant differences in working memory (digit span backwards) in overweight men who were administered either naltrexone (300 mg/day) or placebo. In contrast, animal research suggests naltrexone in rats improves working memory performance (radial arm maze) compared to saline administration (117) and that naltrexone reverses deficits in working memory (radial arm maze) that have been induced by exposure to microwaves (118). However, one study did find the opposite with microwave exposure failing to induce deficits in radial arm maze performance and naltrexone treated rats taking longer to complete the task relative to saline treated animals (119).

In animal studies examining the effects of naltrexone on inhibitory control, naltrexone had no significant effect on delay discounting (inhibiting choice of immediate reward over a larger, delayed reward) when administered alone in rats and mice (120, 121). In contrast, naltrexone has been shown to improve inhibitory control in a rat gambling task (by inhibiting preference for immediate gain at the expense of reduced net gain) in animals that made more suboptimal choices at baseline (122). Additionally, naltrexone pre-treatment improved morphine-induced decrements in impulsive choice (120). Similarly, naloxone (a drug which is used clinically for acute opioid overdose and is a non-selective opioid antagonist which, like naltrexone, blocks μ-opioid receptors with greatest affinity) attenuates drug-induced inhibitory control deficits (five-choice serial reaction time task) in rats (123). In humans, the acute effect of naltrexone (50 mg) on inhibitory control (inhibiting choice of immediate reward over a larger, delayed reward) has been investigated in abstinent alcoholics and healthy controls. Naltrexone did not improve impulsive choice reliably across abstinent alcoholic participants, but performance was instead dependent on personality. Across both abstinent alcoholics and healthy controls, those with greater external locus of control made fewer impulsive choices on naltrexone and the opposite was true for individuals with greater internal locus of control (124). As perceptions of control may be influenced by tonic frontal dopamine (125) and frontal dopaminergic tone may account for individual differences in impulsive choice (126) it is interesting to note here that previous evidence suggests that the opioid system appears to have a role in modulating dopamine tone (127).

Research regarding the effect of naltrexone on cognitive flexibility has been mixed. A study in rats suggests that aged relative to young rats have impaired flexibility on an attentional set-shifting task (extradimensional shifting) and that this age-related impairment was reversed by naltrexone while there was no effect of naltrexone on the performance of younger rats (128). In humans, no significant difference in cognitive flexibility (Color Trails task) was found between abstinent heroin abusers receiving naltrexone therapy and healthy controls whereas buprenorphine-maintained patients showed impairments in cognitive flexibility (129) while, an earlier study in overweight men suggested there was no significant effect of receiving high dose naltrexone (300 mg/day) on cognitive flexibility (Trails B) after 8 weeks of treatment compared to placebo (116).

## Pharmacotherapies for Opioid Use Disorder

### Methadone

As a μ-opioid receptor agonist that also has antagonist properties at the glutamatergic NMDA receptor, methadone is used clinically as an analgesic and is used in OUD where it may be used in long-term maintenance therapy or to manage withdrawal during detoxification (130). Several studies have investigated executive function in patients receiving methadone maintenance therapy. Studies tend to differ in terms of the methadone dose and duration of treatment as well as by comparator i.e., healthy controls with no history of substance abuse, former opioid abusers not in methadone maintenance therapy, or within subject comparisons such as pre-/during therapy and peak/trough concentration following dosing (for studies comparing methadone maintenance therapy with buprenorphine see next section). These differences may account for some equivocal findings described below.

Studies have shown that methadone maintenance therapy is associated with poorer working memory. For instance, those who had been on short-term (at least 30 days) or long-term (at least 6 months) methadone maintenance scored in the lower portion of the normal range for working memory (letter-digit ordering) based on normative test data (131). Working memory (letter number sequencing) was also worse in those in methadone maintenance therapy (mean duration of treatment: 38.66 months; mean dose of methadone: 83.82 mg/day) compared to abstinent heroin abusers although this difference only approached significance (132). Methadone users (mean duration of treatment: 41.48 months) also had significantly worse working memory (2-back task) compared to healthy controls (133). While in another study using a within-subject design working memory (n-back task and modified Sternberg task) was assessed in methadone-maintained patients (mean duration of treatment: 48.9 months; mean dose of methadone: 97.5 mg/day) at approximately 120 min and 26 h after dosing (to coincide with peak and trough methadone concentrations). While there were no differences on the modified Sternberg task, *n*-back performance was slower when testing time coincided with peak methadone concentration. In addition, higher doses of methadone were associated with decreased n-back hit rate (134). However, some studies have found no significant differences in working memory when comparing methadone-maintained patients with healthy controls with no history of substance abuse (135) or with abstinent former opioid abusers (136). The average doses of methadone used in these two studies was 15.14 and 67.2 mg/day, respectively. Taken together it appears that methadone may impair working memory on certain tasks and when higher doses are taken. However, more studies are needed that take into account baseline cognitive performance levels.

Two studies (described above) assessed the effects of methadone maintenance therapy on inhibitory control (132, 133). These studies found that methadone maintenance was associated with poorer inhibitory control (five-digit test) compared to abstinent heroin abusers (132) and poorer inhibitory control (stop-signal task) when compared to healthy controls (133). In another study however, no correlations between dose or duration of methadone maintenance therapy were found in patients where the mean duration of treatment was 8.6 years and the mean dose was 124.2 mg/day (137). Perhaps the longer duration of treatment lead to tolerance of cognitive effects in some participants. Surprisingly, opposite findings have been observed with better inhibitory control (stop-signal task) found in methadone maintenance therapy compared to abstinent opiate dependent participants (138). In their study, Liao et al. (138) found that stop-signal reaction time was significantly shorter in methadone-maintained participants compared to abstinent participants and was no different when compared to healthy controls. Another study comparing methadone-maintained patients and healthy controls stratified patients by duration of treatment (short term: <12 months or long term: ≥12 months) and by dose (low dose: <80 mg/day or high dose: ≥80 mg/day). This study found that healthy participants made more inhibitory errors (errors of commission) on a continuous performance task compared to short term and low dose methadone-maintained patients (139). However, short term and low dose methadone-maintained patients also had the slowest reaction times on the task and the methadone group tended to have poorer sustained attention than healthy controls assessed on the same task. Therefore, the lower number of inhibitory errors found in short term and low dose methadone-treated patients could be due to general task disengagement in this group.

Most studies investigating the effects of methadone maintenance therapy on cognitive flexibility have demonstrated that treatment is associated with impaired flexibility. Those who had been on short-term (at least 30 days) or long-term (at least 6 months) methadone maintenance scored in the lower portion of the normal range for cognitive flexibility (trail making test) based on normative test data (131). In addition, worse cognitive flexibility assessed using a range of cognitive flexibility tasks (trail making test, oral trails, Wisconsin Card Sorting test, switching of attention task) has been reported in methadone-maintained patients compared to abstinent opioid abusers (132, 136) and healthy controls (135, 140, 141). One study found no improvement in cognitive flexibility (trail making test) when comparing opiate dependent participants at baseline and again following 2 months on methadone maintenance therapy (142) and surprisingly, in one study improved flexibility (trail making test) was found as methadone dose increased (134). A further study compared former opiate dependent participants that had been medication free for 10 years with those whom had been on methadone maintenance for the past 10 years (143). This study demonstrated that methadone maintenance was associated with a selective flexibility deficit. While both groups were able to acquire and reverse information about positive and negative outcomes under neutral conditions, Levy-Gigi et al. (143) found that the methadone-maintained group were impaired at reversing positive outcomes when these were presented in a drug-related context.

### Buprenorphine

As a non-selective, mixed agonist-antagonist at opioid receptors (partial agonist at μ-opioid receptor, antagonist at κ- and δ-opioid receptors as well as weak partial agonist at nociception receptors) buprenorphine is used as an analgesic as well as to help manage withdrawal symptoms during opioid detoxification. During detoxification, buprenorphine may be used as short or long-term opioid replacement therapy (for longer-term use it is often combined with the pure opioid antagonist naloxone) and it appears to have similar clinical effectiveness to methadone at managing opioid withdrawal (144).

Few studies have investigated the effects of buprenorphine on executive function. One study investigating the impact of different doses on working memory administered buprenorphine/naloxone to opioid dependent patients at a starting dose of 8 mg/2 mg going up to 16 mg/4 mg and then 32 mg/8 mg with 7–10 days at each dose. This study found that there was no impairment in working memory (N-back task) as the dose increased four-fold (145). However, poorer working memory (Letter-Number Sequencing task, Paced Auditory Serial Addition task) has previously been found in opioid-dependent patients treated with buprenorphine/naloxone compared to healthy controls (146, 147). Rapeli et al. (147) also compare buprenorphine/naloxone treated patients with methadone-maintained patients at several time points (1. 2months, 2. 6–9months and 3. 12–17months after starting substitution therapy) and show that for one of the working memory tasks (Letter-Number Sequencing task) the buprenorphine/naloxone treated group improved between the second and third time points while the methadone treated groups performance remained stable across time. Working memory (digit span backwards) was however not found to be significantly different between patients on either buprenorphine (mean dose: 10.6 mg/day) or methadone (mean dose: 82.7 mg/day) maintenance therapy (mean duration of treatment 48 months across both maintenance therapies) or between these patients (combined in to one group) and healthy controls in a study from another group (148).

Very few studies have assessed the effect of buprenorphine on inhibitory control. One study already mentioned in this section above (148) found that opiate-dependent patients on either buprenorphine and methadone maintenance therapy didn't differ in inhibitory control (Haylings Sentence Completion test) but that when compared to healthy controls these patients (combined in to one group) performed significantly worse. However, another study comparing buprenorphine-maintained opioid dependent patients (mean duration of therapy: 5.4 years; mean dose: 9 mg/day) with both methadone-maintained patients (mean duration of therapy: 8.3 years; mean dose: 66 mg/day) and healthy non-opiate dependent controls found that the buprenorphine treated group performed better than the methadone treated group and no different from controls on the Iowa gambling task (149). The Iowa gambling task is traditionally considered a decision-making task but to perform well on the task it requires the ability to inhibit selection of decks that provide higher immediate gains but long-term losses (150). While, Haylings Sentence Completion test involves inhibition of sensible words that could be used to complete sentences (151). While inhibition is required by both tasks the Iowa gambling task is less semantic and the differing task demands and neural underpinnings may account for the differing findings from these studies.

Several studies have assessed the effect of buprenorphine on cognitive flexibility. Two studies already mentioned in this section above also included an assessment of cognitive flexibility (145, 149). One of these studies did not find impairments in cognitive flexibility (trail making task) with a four-fold increase in the dose of buprenorphine/naloxone given to opioid dependent patients (145). However, in the other study mentioned buprenorphine-maintained patients made fewer perseverative errors on the Wisconsin Card Sorting task compared to methadone-maintained patients with their performance falling somewhere between the group treated with methadone and healthy controls (149). In other studies, a within-subject design found that intravenous infusion of 0.6 mg of buprenorphine to healthy males over 150 min resulted in a significant deterioration in cognitive flexibility (trail making test) compared to a drug-free baseline assessment (152). Studies comparing opioid dependent patients on buprenorphine to healthy controls assessing cognitive flexibility (trail making test, color trails task) have tended to find that the treated patients perform less well than healthy control (129, 153). However, in tasks comparing the cognitive flexibility of buprenorphine and methadone-treated opioid dependent patients two studies failed to find a significant difference in cognitive flexibility in direct contrast to Pirastu et al. (149) (154, 155). While maintenance therapy doses and durations of treatment across these studies were similar, these two latter studies used the trail making test while the Wisconsin Card Sorting task was used by Pirastu et al. (149). The different cognitive demands of these tasks may help explain the differences seen across these studies. In the trail making test participants are required to shift backwards and forwards between numbers and letters in a predictable manner (156). While in contrast, in the Wisconsin Card Sorting task participants are unaware of what shifts will be required when task rules change and must work these out for themselves using feedback (157).

### Lofexidine

Approved for the management of acute opioid detoxification in the United Kingdom in 1994 and more recently by the Food and Drug Administration in the United States in 2018, lofexidine is an α_2A_ adrenergic receptor agonist that has historically been used to reduce blood pressure and is now used to alleviate opioid withdrawal symptoms (158). To the best of our knowledge, there has not been any published research investigating lofexidine's effects on executive function. Studies examining general cognitive effects of lofexidine are also scarce. However, one report in 14 opioid dependent participants, suggests there may be a dose-related deterioration in simple reaction time, continuous performance, procedural memory, and mathematical processing when lofexidine is added to methadone maintenance therapy (159). Nevertheless, other α_2A_ adrenergic receptor agonists have been shown to selectively improve prefrontal cortex mediated cognitive functions (160).

## Discussion

The aim of this review was to provide a brief narrative overview of the evidence for effects of some of the most commonly approved and prescribed pharmacotherapies for TUD, AUD, and OUD on the three core executive functions (working memory, inhibitory control and cognitive flexibility). Enhancement of executive function is likely to be an important target for the treatment of substance use disorders and may contribute to clinical efficacy of existing medications since executive dysfunction is thought to contribute to poor treatment adherence, worse clinical outcomes and relapse (13–19). However, for most of the approved pharmacotherapies reviewed it was difficult to draw firm conclusions regarding effects on executive function. This is due to a surprising lack of well-powered empirical research evaluating the effects of pharmacotherapy on executive function, and because of the extent of contradictory findings. A similar conclusion was made by a recent systematic review of the general cognitive effects of existing pharmacotherapy (23).

Both hypo- and hyperdopaminergic states have been postulated to account for various addiction phenomenon in the absence and presence of drug cues (161). Positron-emission tomography (PET) studies in substance abusing populations suggest that there are decreases in both dopamine release and dopamine D_2_ receptors (162, 163). Indeed, the dopamine hypothesis of drug addiction (164) implicates a long-lasting hypodopaminergic state throughout the addiction cycle including persistence of this state in withdrawal. For example, PET imaging with a high affinity dopamine D_2/3_ receptor radioligand has established that there is a smaller amphetamine-induced dopamine release in the cortex and midbrain of abstinent alcoholics than in healthy controls (165). Many of the pharmacotherapies reviewed here have direct or indirect effects on dopamine levels. In line with the inverted “U” shaped dose response curve for dopamine effects on executive function (62) drugs that enhance dopamine levels in individuals with a low baseline level of dopamine would be expected to enhance executive function while potentially impairing the performance of individuals with a higher dopaminergic starting point. Mixed findings in the current review may be attributable to differing dopaminergic baselines. In this regard, medicated substance dependent patients with lower baseline dopamine and greater cognitive impairments may receive greater cognitive benefit than less cognitively impaired patients with a higher dopaminergic baseline. While it may be more difficult to demonstrate cognitive improvements in healthy participants or there may be paradoxical impairment in performance.

Substance use disorder pharmacotherapies have been shown to be efficacious however they do not work for everyone. Identifying for whom they do, and do not, work is an important unmet clinical need. While it is evident that executive dysfunction is observed during early abstinence which may contribute to relapse (20–22) much more work is required in order to determine whether a drugs positive effects on executive function are predictive of positive cessation outcomes. Previous PET imaging studies with a high affinity dopamine D_2/3_ receptor radioligand have suggested that the extent to which methylphenidate induces increases in dopamine are predictive of relapse and response to behavioral and psychological treatments in methamphetamine and cocaine abusers (166, 167). Future research should investigate whether clinical effectiveness of pharmacotherapy (i.e., sustained cessation) is related to individual differences in the ability of the drugs to improve cognitive function and whether this is associated with baseline differences or changes in dopamine levels.

Existing and novel cognitive enhancers may be beneficial for substance abuse disorders and studies investigating effects of cognitive enhancers are on-going (7). Whether it is existing pharmacotherapies being evaluated for their effects on cognition, or novel cognitive enhancers being evaluated for the potential to improve executive function and clinical outcomes in substance dependent populations, it is important to consider how cognition will be assessed. The current narrative review illustrates that even when the number of studies assessing different components of executive function are small, a wide variety of tasks and outcome measures are used which can make cross-study comparisons difficult. Future studies should carefully consider which tasks are best suited to assess relevant cognitive functions. Future work should also consider the potential cognitive enhancers mechanism of action and abuse potential. For example, modafinil is a promising cognitive enhancer but it's addictive potential has been illustrated in studies examining effects on behavioral sensitization and conditioned place preference (168).

An alternative approach to try and improve executive function in addiction has been with cognitive training most notably working memory training and inhibitory control training. Training of working memory has been found to improve working memory performance and reduce subsequent drug use in methadone-maintained patients and problem drinkers compared to control conditions (169, 170). Similarly, inhibitory control training using an alcohol-related Go/NoGo task has previously been found to reduce post-training alcohol consumption as effectively as a Brief Alcohol Intervention (171). Reduced drug use post-training suggests that interventions based on these types of training procedure may improve clinical outcomes and further supports the targeting of executive function in addiction. However, future studies should consider whether pharmacotherapy could compliment and even facilitate such training. Inhibitory control training, for example, may work via the devaluation of reward-related stimuli (172) and given that some of the drugs reviewed here e.g., varenicline, disulfiram, nalmefene, and naltrexone may devalue substances of abuse (either by reducing the positive rewarding effects of substances or by pairing them with an unpleasant reaction) it would be interesting to see whether these drugs are able to facilitate inhibitory control training and improve dependent populations control over substance use in real-world settings.

In this review we have examined the evidence for executive function enhancement by commonly prescribed, labeled pharmacotherapy for TUD, AUD, and OUD as any such enhancement may contribute to clinical efficacy. However, it should be noted that the act of detoxification might itself be expected to improve executive function. Future studies should include appropriate controls or take this variable in to account when estimating the cognitive effects of medications used to assist detoxification maintenance. While a potential strength of this review is that it has evaluated the cognitive impact of only those medications with a high degree of evidence for efficacy in treating TUD, AUD, and OUD this does mean that we may have missed important trends in findings with those medications that are used off-label to treat these disorders (e.g., topiramate). In addition, this review excluded off-label pharmacotherapy for other substance use disorders such as the stimulants cocaine and methamphetamine. These disorders are persistent public health problems for which there are no approved pharmacotherapy options (173, 174). While the relative lack of evidence for consistent and positive pharmacotherapy effects, coupled with a wide-range of off-label prescribing practices lead us to exclude such research this too may have led to missing important trends in findings and consequently limited our discussion.

## Conclusions

There are several efficacious pharmacotherapy options available for TUD, AUD, and OUD. Evidence is limited and conflicting regarding whether they can improve executive function in dependent populations. It should be noted that baseline differences in dopamine and performance may contribute to an explanation for why inconsistent findings exist. So far, strategies aimed at enhancing cognition to help with improving cessation rates in dependent populations have not been successfully implemented in the clinic. However, there has been limited research conducted in this area and cognitive enhancement remains a potential strategy that is worth exploring further. The issue of abuse liability of drugs that can be cognitive enhancers needs to be taken in to consideration when designing such studies. Moreover, studies should now move beyond simply assessing cognitive effects in order to establish whether an improved cognitive response is related to clinical efficacy and if this is also associated with baseline or changes in dopamine. This approach may assist future personalized medicine strategies.

## Author Contributions

All authors contributed to conception of this review, KB drafted the manuscript and all authors contributed to manuscript revision, read and approved the submitted version.

### Conflict of Interest Statement

BLF has obtained funding from Pfizer GRAND Awards (with donation of varenicline) and a grant from Alkermes (with donation of Vivitrol) and multiple other grants not related to the present work. The remaining author declares that the research was conducted in the absence of any commercial or financial relationships that could be construed as a potential conflict of interest.
